# Assessment of the Relationship Between Bioexclusion Practices Applied in Wean-to-Harvest Sites and PRRS Outbreaks

**DOI:** 10.3390/vetsci12101000

**Published:** 2025-10-16

**Authors:** Mariah Musskopf, Tina Peterson, Isadora Machado, Thinh Tran Pham Tien, Elly Kirwa, Daniel Carnevale de Almeida Moraes, Guilherme Cezar, Mafalda Mil-Homens, Peng Li, Elisa De Conti, Ana Paula Poeta Silva, Derald J. Holtkamp, Daniel C. L. Linhares, Gustavo S. Silva

**Affiliations:** Department of Veterinary Diagnostic and Production Animal Medicine, College of Veterinary Medicine, Iowa State University, Ames, IA 50011, USA; mariahnm@iastate.edu (M.M.);

**Keywords:** porcine reproductive and respiratory syndrome, swine, biosecurity, bioexclusion, wean-to-harvest, surveillance

## Abstract

**Simple Summary:**

Porcine reproductive and respiratory syndrome (PRRS) is a major disease affecting pigs in the United States (U.S.), causing economic losses and threatening animal health. It can spread between sites through infected animals or contaminated people, vehicles, and equipment. This study followed groups of pigs at 95 swine sites in the Midwest from nurseries, finishers, and wean-to-finish facilities to determine farm practices associated with PRRS outbreaks. Oral fluids were collected every four weeks to detect infection, and sites were surveyed once about biosecurity practices. The results indicate that transporting pigs of unknown PRRS status, using rendering, and employees living with people who worked on other sites were associated with increased incidence of PRRS outbreaks. In contrast, having a designated parking area, using equipment only for one farm, and requiring overnight breaks for workers who visit multiple sites reduced the odds of outbreak in the univariate analysis. In the multivariable model, sites located within one mile of another swine site also faced higher odds. These results can help pig farmers and the swine industry focus on practices that lower the odds of disease and improve animal health.

**Abstract:**

Porcine reproductive and respiratory syndrome virus (PRRSV) is a significant cause of economic loss in the swine industry, yet its control remains challenging in wean-to-harvest sites. This prospective observational study followed 95 wean-to-harvest sites across six U.S. states for one production cycle. Sites were required to be PRRSV-negative or vaccinated with a modified live virus (MLV) and free of major coronaviruses. Outbreaks were defined as RT-qPCR-positive in unvaccinated sites or detection of ORF5 sequences distinct from the MLV strain. Biosecurity data were collected through a survey, and oral fluids were tested every four weeks. PRRS outbreaks occurred in 14/42 nurseries (33.3%), 8/12 wean-to-finish (66.7%), and 35/41 finishers (82.4%), with lineage 1C.5 most frequently detected. In univariate models, higher odds of outbreaks were associated with transporting pigs of unknown status (OR 9.80, 1.73–55.37), rendering (OR 6.47, 1.62–25.84), and employee cohabitation (OR 6.15, 1.51–25.09). Protective factors included exclusive pumping equipment (OR 0.07, 0.01–0.43) and overnight downtime for multi-site workers (OR 0.15, 0.04–0.56). In multivariable models, finisher sites (OR 17.47, 2.44–125.19) and greater swine site density within one mile (OR 1.62, 1.09–2.41) significantly increased outbreak odds. These results support targeted biosecurity practices, helping farmers and the swine industry reduce PRRS outbreaks.

## 1. Introduction

Porcine reproductive and respiratory syndrome (PRRS) is caused by the porcine reproductive and respiratory syndrome virus (PRRSV), that belongs to the virus species within the *Arteriviridae* family, with PRRSV-2 being the predominant species in North America [1,2,3,4]. The acute PRRSV infection in nursery or grower pigs typically presents with anorexia, lethargy, red discoloration of the skin, labored or rapid breathing, intermittent coughing, rough hair coats, decreased average daily gain, and increased mortality rates ranging from 12% to 20% [5]. More recent reports indicate rates as high as 60–70% throughout swine production [6]. The clinical signs significantly contribute to the economic burden of PRRSV in the United States (U.S.). The financial impact of PRRSV during the wean-to-harvest phase is substantial. More recently, the annual losses have been estimated at $1.2 billion, with approximately 68% of these losses attributed to the wean-to-harvest phase, equivalent to about $4.67 per pig marketed in reduced net revenue [7].

Biosecurity is a set of management and physical practices designed to prevent the introduction and spread of pathogens within and between animal populations [8]. It encompasses three components: bioexclusion (preventing pathogens from entering), biocontainment (preventing the spread of pathogens from farm to farm), and biomanagement (reducing the impact of pathogens already present) [9,10].

Effective biosecurity requires an understanding of disease epidemiology, including transmission routes, pathogen survival in the environment, and the role of fomites and vectors [11]. Among the components, bioexclusion is particularly important, focusing on preventing infection from external sources such as incoming animals, people, vehicles, equipment, and feed [12,13]. Key practices aim to limit direct and indirect contact with contaminated fomites or mechanical vectors, including personnel, transport vehicles, equipment, and third-party services like repair and rendering [14,15].

While sow sites frequently have well-established biosecurity protocols, such as bench entry systems and shower-in procedures, knowledge about bioexclusion practices in wean-to-harvest sites remains limited [16]. This gap is notable, given that growing pigs represent the majority of U.S. production and play a pivotal role in maintaining and disseminating pathogens. Studies on sow sites have identified risk factors such as the frequency of risk events (including animal movements, supply deliveries, people entry, contact with other animals, and potential transmission through air or water), nearby swine density, farm characteristics, and operational connections to other sites. Additional factors associated with increased disease risk include staff turnover, trailer sharing, proximity to roads, and production type [13].

Despite decades of research and substantial industry investments in biosecurity, efforts to eliminate endemic PRRSV have yielded inconsistent results, particularly during the growing phase of production [17]. These challenges are largely attributed to the complex nature of disease transmission, the variability in biosecurity implementation, and the difficulty of sustaining pathogen exclusion across diverse wean-to-harvest systems [13,18,19]. Growing pigs can play a crucial role in maintaining, amplifying, and disseminating pathogens throughout the production system. Frequent pig movements, co-mingling from multiple sources, and diverse site management practices can create ideal conditions for the persistence and spread of infectious agents such as PRRSV. Data from the Swine Disease Reporting System (SDRS) has shown that increases in PRRSV PCR positivity in wean-to-harvest sites often precede similar increases in sow herds, suggesting the critical role of this phase in shaping transmission dynamics [20]. Yet, the available literature on biosecurity practices has thus far focused almost exclusively on sow herds, leaving an important gap in evidence for wean-to-harvest production.

Despite the economic and epidemiological importance of the wean-to-harvest phase, there is a lack of knowledge regarding which bioexclusion practices are most effective for these populations and how they should be assessed. Therefore, the objective of this study was to identify and evaluate the association between specific biosecurity practices and PRRS outbreaks in wean-to-harvest sites.

## 2. Materials and Methods

### 2.1. Study Design and Population

A prospective observational study was conducted in wean-to-harvest pig production sites (nurseries, finishers and wean-to-finish facilities) managed by commercial swine companies across the United States. Participating sites were enrolled at the time of animal placement (around 5 weeks and 11 weeks of age). Groups of pigs were followed throughout a complete production cycle, until pigs were either marketed or transferred to another location. Each site completed a standardized biosecurity assessment to document its practices by the herd veterinarian. PRRSV surveillance data was collected throughout the production cycle to monitor pathogen presence.

### 2.2. Herd Selection and Inclusion Criteria

Participating sites were required to meet the following inclusion criteria:

- Agree to complete a site-specific biosecurity survey;
- Provide farm geolocation;
- Collect oral fluid samples for diagnostic testing throughout the production cycle of a single group of pigs;
- Each flow had to be either PRRSV modified live vaccine (MLV) vaccinated or negative status, i.e., PRRSV wild type PCR negative confirmed by Sanger sequencing, and free from active infection with major enteric coronaviruses (Porcine Epidemic Diarrhea Virus [PEDV], Porcine Deltacoronavirus [PDCoV], and Transmissible Gastroenteritis Virus [TGEV]) confirmed by RT-qPCR multiplex.

In instances where sites used a PRRSV MLV vaccine, vaccination details were recorded, such as date and vaccine type. Herd sizes were measured, and the operational structure was documented, including whether sites functioned as independent systems or as part of an integrated production network.

### 2.3. Site Recruitment

Recruitment of study sites began in September 2023. Sites were recruited using a contact list developed from the 2022 Power Pork Producers network. Companies were contacted based on the list and by convenience with a presentation of the project, and upon agreement to enroll their sites, surveys were electronically distributed, and sample collection was scheduled, with strict confidentiality maintained throughout the process. Sample collection did not occur simultaneously across all facilities; instead, sampling was initiated as farms were recruited in October 2023, with the last sampling conducted at the end of October 2024. Consequently, different sites were sampled at different time frames, reflecting their individual enrollment dates rather than a single coordinated collection period. Sites enrolled in the study were in the Midwest region of the US, which is the densest swine farm region.

### 2.4. Bioexclusion Assessment

Bioexclusion data were collected using a survey. An initial virtual meeting was held with each participant (e.g., veterinarians, production managers, or biosecurity officers) to introduce the study objectives and instructions. Participants completed one 115-question survey per site. Several questions allowed multiple responses, resulting in a greater number of variables than questions. The survey covered 17 categories, which are displayed in Table 1. The survey used in this study is available in File S1 in the Supplementary Materials.

Surveys were submitted electronically to herd veterinarians from the enrolled companies. Follow-up emails and phone calls were implemented to clarify responses and ensure data consistency throughout the study period when needed.

### 2.5. Sample Collection and Surveillance

Oral fluid samples were collected once every four weeks from placement until the end of each phase. At each sampling time point, six ropes were distributed across pens using a zig-zag spatial approach to ensure coverage throughout the barn. Pigs were allowed to chew on 5/8-inch cotton ropes (Skydog Rigging Equipment, Lake in the Hills, IL, USA) for 20–30 min, after which fluid was manually harvested into 50 mL centrifuge tubes (Thermo Fisher Scientific, Pittsburgh, PA, USA) [21]. For diagnostic testing, the six samples obtained per site were pooled in sets of three, resulting in two combined samples per farm per sampling event. Pooling was necessary for logistical and cost considerations, and previous work has demonstrated that pooling oral fluids at this level does not markedly reduce sensitivity for PRRSV detection compared with individual testing [22].

Samples were refrigerated immediately post-collection and shipped to the Iowa State University Veterinary Diagnostic Laboratory (ISU VDL). The oral fluid samples were tested in pools of three.

### 2.6. Diagnostic Testing

The samples were tested for molecular diagnostics at the ISU-VDL, under its procedures and standards, which is accredited by the American Association of Veterinary Laboratory Diagnosticians (AAVLD). All samples were tested using validated commercial RT-qPCR assays to detect North American and European PRRSV strains. Additionally, samples were screened for PEDV, PECoV, and TGEV using multiplex RT-qPCR assays. The samples were considered PRRSV-positive (PED, TGE, PEDCOV)-positive when the cycle threshold (Ct) was <37. Those with Ct values < 34 were submitted for open reading frame 5 (ORF-5), using Sanger sequencing, to distinguish vaccine-like, wild-type, and previously identified strains.

### 2.7. PRRS Outbreak Definition

For unvaccinated sites, an outbreak was defined as the detection of PRRSV RNA by RT-qPCR from oral fluid samples. For sites using modified-live virus (MLV) vaccines, outbreak confirmation required sequencing of the ORF5 gene to differentiate wild-type from vaccine-like viruses. Across the study, 39 outbreaks (70.9%) were confirmed by sequencing and 16 outbreaks (29.1%) were classified based on PCR detection alone at unvaccinated sites.

### 2.8. Statistical Analysis

Descriptive statistics of herd characteristics, such as median and range for site and company size, were calculated. A total of 273 bioexclusion variables were evaluated as potential risk factors for PRRS outbreaks. The outcome of interest was whether a site had at least one positive sampling during the study period, which was classified as a PRRS outbreak.

For the univariate analysis, logistic mixed-effects models were fitted based on the following specification:

logit(*P*(*Y_ij_* = 1)) = *β*_0_ + *β*_1_*X_ij_* + *μ*_*j*_

where *Y*_*ij*_ represented the outbreak status of site *i* within production system *j*, *β*_0_ the intercept, *β*_1_ the effect of the explanatory variable *X*_*ij*_, and *u*_*j*_ a random intercept for the production system to account for clustering of sites within companies.

Variables with a *p*-value < 0.20 in univariate analysis were selected as candidates for the multivariable analysis. The multivariable model was specified as:

logit(*P*(*Y_ij_* = 1)) = *β*_0_ + *β*_1_*X*_1*ij*_ + *β*_2_*X*_2*ij*_ + *β*_n_*X*_n*ij*_ + *μ*_*j*_

where multiple explanatory variables were included simultaneously, and the production system remained as a random effect. Forward stepwise selection was used to refine the model, sequentially removing non-significant terms based on likelihood ratio tests and Akaike Information Criterion (AIC). Model assumptions, including linearity for continuous variables and multicollinearity, were assessed.

All models were fitted in R (version 4.4.1, “Race for Your Life”; R Core Team, 2024) using the lme4 and lmerTest packages [23]. Statistical significance was set at *p* ≤ 0.05, and measures of association were presented as odds ratios (OR) with 95% confidence intervals (CI).

### 2.9. Animal Care and Use

The animal study protocol was approved by Institutional Animal Care and Use Committee IACUC of Iowa State University, IA USA under the protocol IACUC-22-101 and IBC-24-087.

## 3. Results

The dataset included 95 sites from eight companies operating in six states: Iowa (26), Illinois (7), Indiana (15), Minnesota (4), Nebraska (42) and Ohio (1). These sites consisted of 33 (34.7%) nursery sites, 17 (17.9%) finisher sites, and 45 (47.4%) wean-to-finish sites. Nursery sites had sizes ranging between 4819 and 26,016 pigs, with a mean centered around 11,445 pigs (Table 2).

The average number of swine sites within 1-, 3-, and 5-mile radio was consistently higher for PRRSV-positive sites compared to PRRSV-negative sites. Specifically, PRRSV-positive sites had an average of 1.92 neighboring sites within 1 mile, 9.67 within 3 miles, and 20.45 within 5 miles. In contrast, PRRSV-negative sites had numerically lower averages of 1.05, 5.57, and 11.37 sites within the same respective distances.

Finisher sites had the highest PRRS outbreaks at 82.4% (14/17), followed by wean-to-finish sites at 66.7% (30/45) and nursery sites at 33.3% (11/33).

Regarding the PRRSV ORF-5 sequencing, five distinct lineages were identified across sampled sites (Table 3). In nursery sites, lineage L1C.5 was detected twice. Wean-to-finish sites exhibited the greatest diversity, with L1C.5 being the most frequent (13 detections), followed by L1A (5), L5A (3), L1H (2), and L1D (1). Among finisher sites, L1C.5 also predominated (4 detections), with single detections of L1D and L1H. Additionally, 71 samples tested positive for PRRSV by qPCR but did not yield a sequence, which is consistent with previous studies [24], where low viral load (high Ct values) limited sequence recovery. Only two sites experienced introductions of genetically distinct wild-type PRRSV strains, one case involved a co-infection with L1C.5 and L1D, while the other detected different lineages (L1H and L1C.5) at separate time points.

**Figure 1:**
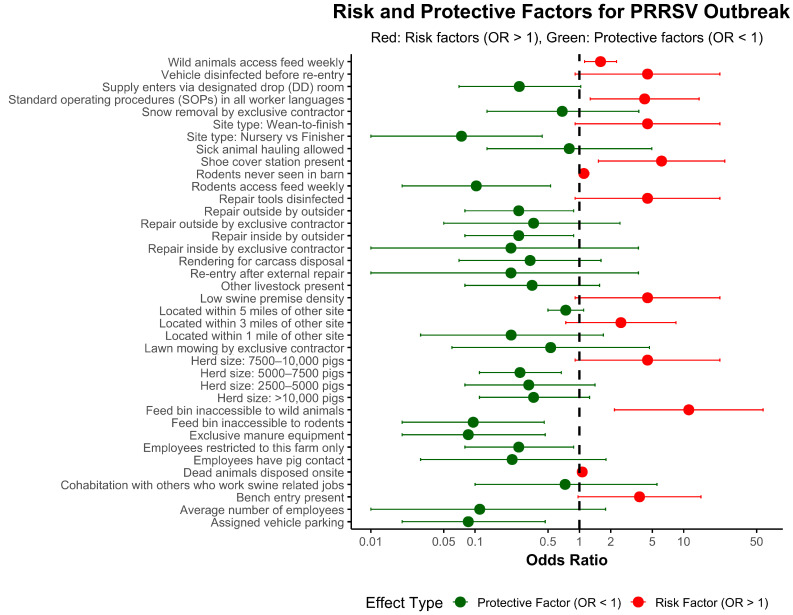
Risk factors associated with PRRS outbreaks. The forest plot displays the odds ratios for various biosecurity and management practices associated with PRRS outbreaks. Practices with odds ratios above 1 indicate increased odds of outbreak (red dots), while those below 1 suggest protective effects (green dots).

The univariate variables with a *p*-value < 0.05 in the univariate analysis are displayed in Figure 1 and Table 4.

Among the risk factors evaluated, natural air entrance, hauling pigs with unknown PRRS status, use of rendering, and cohabitation with others who work swine related jobs were significantly associated with higher odds of PRRS outbreaks, as indicated by red dots and elevated odds ratios in Figure 1. The corresponding values are presented in Table 4. For example, sites where employees cohabitated with others involved in swine-related work (red dot) had 6.15 times higher odds of a PRRS outbreak (95% CI: 1.51–25.09, *p* = 0.011), and those using rendering for carcass disposal (red dot) showed 6.47 times higher odds compared to sites without rendering (95% CI: 1.62–25.84, *p* = 0.008). In contrast, practices such as maintaining a defined parking space (green dot) (OR = 0.07, 95% CI: 0.01–0.35, *p* = 0.001) and using exclusive pumping equipment (green dot) (OR = 0.07, 95% CI: 0.01–0.43, *p* = 0.003) were associated with substantially lower odds of a PRRS outbreak, suggesting a protective effect. The tables describing the univariate results for the variables with *p*-values < 0.20 and the frequency of adoption of bioexclusion practices by site type are presented, respectively, in Tables S1 and S2 in the Supplementary Materials.

**Table 4:** Bioexclusion practices with *p*-value <0.05 associated with sites reporting a PRRS outbreak in the univariate model.

Biosecurity Practices	Levels	Odds Ratio	CI 95%	*p*-Value
Use of rendering as dead pig disposal method	No (Reference)	1		
	Yes	6.47	1.62–25.84	0.008
Natural vs. mechanical ventilation system	Mechanical (Reference)	1		
	Natural	10.88	1.35–87.60	0.025
Pigs hauled without confirmed PRRS status	No (Reference)	1		
	Yes	9.79	1.73–55.37	0.009
Allowing employees to cohabitate with others who work swine related jobs	No (Reference)	1		
	Yes	6.15	1.51–25.09	0.011
Production site type category	Nursery (Reference)	1		
	Finisher	17.47	2.44–125.19	0.004
	Wean-to-finish	8.47	0.85–84.24	0.068
Dedicated vehicle parking area for staff/visitors	No (Reference)	1		
	Yes	0.07	0.01–0.35	0.001
Dedicated manure pumping equipment per site	No (Reference)	1		
	Yes	0.07	0.01–0.43	0.003
Multi-farm employee mandatory overnight downtime	No (Reference)	1		
	Yes	0.15	0.04–0.56	0.004
Rodents never have access to feed bin	Reference			
	Never	0.08	0.02–0.47	0.004
Wild animals never have access to feed bin	Reference			
	Never	0.08	0.02–0.47	0.004
Presence of bench entry	No (Reference)			
	Yes	0.27	0.11–0.67	0.005
Exclusive 3rd party manure handling per site	No (Reference)			
	Yes	0.10	0.02–0.53	0.006
Site located within 1 mile of another swine site	Within 1 mile	1.59	1.12–2.27	0.010
Site located within 5 miles of another swine site	Within 5 miles	1.06	1.01–1.12	0.029

The results of the multivariable logistic regression model identified significant associations between PRRS outbreak and both site type and local swine site density (Table 5). Compared to nursery sites (reference category), finisher sites had significantly higher odds (*p*-value = 0.004) of experiencing a PRRS outbreak, with an odds ratio of 17.47 (95% confidence interval [95% CI]: 2.44–125.19). In contrast, wean-to-finish sites had an odds ratio of 8.47 (95% CI: 0.85–84.24), which was not statistically significant (*p* = 0.068). Additionally, the presence of at least one other swine site within a 1-mile radius was associated with a higher number of outbreaks (*p* = 0.018), with an odds ratio of 1.62 (95% CI: 1.09–2.41).

**Table 5:** Farm type and bioexclusion practices associated with sites reporting a PRRS outbreak.

Biosecurity Practices	Levels	Odds Ratio	CI 95%	*p*-Value
Site type	Nursery (Reference)	1		
	Finisher	17.47	2.44–125.19	0.004
	Wean-to-finish	8.47	0.85–84.24	0.068
Number of sites in 1-mile radius		1.62	1.09–2.41	0.018

Note: CI denotes confidence interval, representing the range within which the true parameter value is expected to lie with 95% certainty. Reference indicates the base category against which other groups were compared in the regression model.

## 4. Discussion

PRRSV remains one of the most economically and epidemiologically impactful swine pathogens worldwide [7]. The present study contributes to this body of evidence by demonstrating that production type, local swine site density, and specific bioexclusion practices are associated with outbreaks in wean-to-harvest sites.

At the time recruitment began, the general level of PRRSV circulation in wean-to-harvest systems varied across regions. According to the Swine Disease Reporting System (SDRS) [25] monitoring data for October 2023, Iowa reported 59.8% positive submissions, which was more than 3 standard deviations (STDs) below its historical baseline. Nebraska and Indiana were also classified as 3+ STDs below baseline, with 31.3% and 44.8% positive submissions, respectively. In contrast, Illinois and Ohio showed elevated detection, with 29.5% and 9.2% positives, corresponding to 3+ STDs and 2–3 STDs above baseline, respectively. Minnesota remained closer to expected levels, with 39.7% positives, classified as within ±2 STDs of baseline. These findings indicate that, while PRRSV remained endemic across major swine-producing states, overall activity in October 2023 was lower than expected in several Midwestern states, with localized areas of increased detection.

Finisher sites had the highest outbreak rate (82.4%), followed by wean-to-finish (66.7%) and nursery sites (33.3%). This pattern is likely multifactorial, including differences in pig age, population dynamics, phase length, and site-level biosecurity practices. Finisher barns often receive pigs from multiple sources, increasing the probability of introducing infected animals [17,26]. They also tend to have less stringent biosecurity protocols compared to breeding herds, partly because they are positioned at the end of the production chain, where the perceived risk to breeding stock is lower. Furthermore, finishers frequently experience higher rates of pig movement (marketing, transport to slaughter) and more frequent visitor access, both of which can introduce or spread pathogens. These observations are consistent with SDRS reports, which show that PRRSV detection in wean-to-market sites, including finishers, contributed disproportionately to outbreak signals [25]. Recently published research further highlights that growing pig sites represent the majority of U.S. production sites (>86%) and exhibit wide variability in biosecurity implementation [16]. Taken together, these findings suggest that the elevated outbreak frequency observed in finisher and wean-to-finish sites is shaped not only by production and animal flow dynamics but also by heterogeneous adoption of biosecurity practices, highlighting the need for targeted control practices in these settings.

Regarding the sequencing part of the study, analysis of RT-qPCR-positive samples demonstrated that multiple PRRSV lineages were circulating across participating sites (Table 3). The detection of multiple PRRSV lineages across sites reflects the extensive genetic and antigenic diversity of the virus in U.S. production systems. Such diversity complicates outbreak investigations and vaccine-based control, as the frequent emergence and sequential turnover of sub-lineages and variants can undermine immunity and facilitate the spread of novel strains [27]. Regarding the positives that did not yield a sequence, the outcome is not unusual, as sequencing requires a higher pathogen load than PCR, and samples with high Ct values often cannot be reliably sequenced [24].

Local swine site density, particularly the presence of neighboring swine sites within a 1-mile radius, was also strongly associated with outbreak risk. Higher density increases the likelihood of airborne virus drift, shared transport routes, and personnel overlap [14,15]. PRRSV survival in aerosols and mechanical spread via vehicles and insects further amplifies this risk. Previous work by Corzo et al. (2010) [28] and Silva et al. (2019) [13] similarly found that sites located in high-density regions had significantly greater odds of outbreaks, emphasizing that geography is not merely a background variable but an actionable epidemiological risk factor. Unlike those studies, which focused solely on sow farm sites, our findings suggest that higher local swine site density is associated with an increased risk of PRRSV detection during the wean-to-harvest phase.

Several bioexclusion practices emerged as high-impact risk factors for PRRS outbreaks. Rendering carcasses off-site, allowing unfiltered natural air entry, and transporting pigs with unknown PRRS status were all associated with higher odds of infection. Each of these practices represents a potential breach in structural or operational biosecurity. For example, rendering trucks may visit multiple sites and carry pathogens on wheels or equipment, a concern supported by recent outbreak investigations of *Actinobacillus pleuropneumoniae* serotype 15 in central Iowa, where third-party rendering contractors were identified as a significant biosecurity risk within a 40 km radius of index cases [29]. Similarly, unfiltered air entry can increase vulnerability to airborne PRRSV incursion in dense swine regions, although aerosol transmission remains an area requiring further investigation. Successful airborne spread depends on a cascade of probabilistic events, including virus shedding at sufficient concentration, airborne stability, environmental persistence despite dilution, and eventual deposition or entry into susceptible animals, many of which are not yet fully understood [30]. Finally, moving pigs without a verified health status creates a direct route for virus introduction, while cohabitation with other swine-related workers likely reflects reduced adherence to biosecurity protocols overall, serving as a proxy for less controlled environments and increased cross-site exposure.

Conversely, certain practices demonstrated a protective effect. Establishing defined parking areas appears to reduce cross-contamination by limiting vehicle circulation and contact zones, thereby decreasing the likelihood of fomite-mediated transmission. Similarly, the use of exclusive manure pumping equipment reduces the sharing of potentially contaminated tools. These practices are especially relevant in light of recent evidence showing that manure pumping and proximity to recently manured fields significantly increased the odds of PRRS outbreaks, with odds ratios of 3.38 (95% CI: 1.86–6.11) for pumping events and up to 4.09 for nearby applications within 1.6 km [31]. The study further highlights that when multiple third-party pumping contractors serve swine operations in high-density regions, variability in biosecurity practices and increased movement of personnel and equipment across farms may erode control practices. In this context, employing dedicated equipment and clearly defined site boundaries acts as a strategic barrier, mitigating indirect exposure risks associated with manure handling and landscape-level contact networks.

Overnight downtime for employees working across multiple sites allows for a potential “wash-out” period, reducing the chance of pathogen carriage. These practices align with industry recommendations for pragmatic, cost-effective interventions [16] and suggest that small, enforceable changes in daily operations can yield measurable reductions in risk. This recommendation is supported by experimental evidence demonstrating the role of personnel in the mechanical transmission of PRRSV. Dee et al. (2003) [32] demonstrated that individuals who frequently came into contact with infected pigs carried viable virus on their hands, boots, and coveralls, and that transmission to naive pigs occurred in all tested cases following such exposure. Similarly, Pitkin et al. (2009) [14] demonstrated that PRRSV could be transported between sites via contaminated personnel and clothing under field-simulated conditions, even after short travel times. These studies highlight that both direct contact and residual contamination on people and equipment can serve as efficient routes of viral dissemination. The detection of PRRSV on fomites such as cable snares and bleeding equipment further reinforces the potential for indirect transmission. Together, these findings underscore that practical interventions, such as overnight downtime, site-dedicated clothing, and limiting cross-site personnel movement, are not only cost-effective but grounded in strong experimental and field-based evidence.

These findings have direct implications for the control of PRRSV. As U.S. swine production continues to shift toward larger, multi-site systems [25], intervention efforts should focus on finishers in dense production areas, where the combination of high pig movement, environmental exposure, and local connectivity creates ideal conditions for virus introduction.

This study has some limitations that should be acknowledged. First, differences in production phase length introduced a potential time-related confounding effect, since finisher sites were followed for shorter periods than wean-to-finish sites. Although all herds were monitored throughout a complete production cycle, the unequal observation time may have influenced outbreak detection, particularly between nurseries, finishers, and wean-to-finish facilities. Relationships between nursery and finisher sites within the same company were not explicitly tracked, and it is possible that movement connections or shared resources among them could have affected the observed outbreak patterns. Second, as with most observational field studies, variation in the underlying and unmeasured risk of exposure across sites cannot be ruled out. The true probability of encountering PRRSV differs by geography, swine density, and unobserved local management, which may have biased associations. Future work could address this by incorporating dynamic measures of regional PRRSV activity or network-level contact structures to better approximate heterogeneous exposure risks.

Another limitation is the reliance on self-reported survey data, which may have led to overestimation of compliance with biosecurity practices. Similar concerns have been documented by Johnson et al. (2025) [33], who emphasized that reported practices frequently diverge from on-site implementation. The absence of external validation, such as direct observation or electronic auditing, restricts confidence in the accuracy of reported measures. Future studies should consider triangulating survey data with camera-based auditing, digital movement logs, or other objective measures to mitigate reporting bias.

Finally, although the inclusion of 95 sites across six states and eight companies strengthened generalizability, the high number of variables assessed (273) relative to the sample size limited statistical power and increased the risk of spurious findings. To reduce the chance of overfitting, variables were screened using both statistical and biological criteria before inclusion in multivariable models. Nonetheless, the possibility of type I error remains. Future work with larger sample sizes, penalized regression techniques, or external validation datasets would help strengthen inference and reduce the potential for spurious associations.

In conclusion, this study demonstrated that production type, site density, and specific bioexclusion practices were associated with PRRS outbreaks in wean-to-harvest sites. The results reinforce the epidemiological understanding that finishers in dense regions face the greatest threat and that targeted, practical biosecurity practices can mitigate this risk. These findings provide evidence to guide site-specific prevention strategies and support future hypothesis-driven research aimed at reducing the burden of PRRSV in growing pig populations. As a result, even sites with strong bioexclusion protocols may still experience outbreaks if exposure pressure is high, while farms in lower-risk environments may remain negative despite less stringent measures. Although PRRSV incidence is not a perfect indicator of biosecurity efficacy, these findings provide evidence to guide site-specific prevention strategies and underscore the importance of framing biosecurity as a risk-reduction tool rather than a guarantee. Taken together, the study supports future hypothesis-driven research and continued refinement of biosecurity protocols aimed at reducing the burden of PRRSV in wean-to-harvest populations.

## Figures and Tables

**Table 1:** Categories included in the standardized biosecurity survey.

Category	Description/Examples
General information	Baseline farm data collected for context
Site characteristics/biosecurity protocols	Infrastructure, barriers, and standard procedures
Animal transportation	Movements of pigs between sites
Dead pig removal	Processes for mortality disposal
Feed/feed ingredients	Delivery and handling of feed sources
Propane/fuel delivery	Entry of fuel or propane onto the site
Garbage collection	Waste removal procedures
New tools/supply delivery	Direct supply entry from external sources
Transferred tools/supply delivery from other sites	Movement of equipment or supplies between farms
On-farm employee movement	Internal staff movement patterns
Repair/service personnel	Entry of maintenance and service providers
Veterinarians/vendors/visitors/off-farm personnel	External professional and visitor entry
Pork/food entry	Introduction of pork or other food products onto the farm
Manure removal	Transportation and disposal of manure
Other animal entry	Contact or entry of non-swine animals
Air entry	Potential airborne pathogen introduction
Water entry	Potential waterborne pathogen introduction

**Table 2:** Distribution of site sizes by production type.

Production Type	Median Size	Minimum Size	Maximum Size
Nursery	15,417	4819	26,016
Finisher	17,390	2400	32,380
Wean-to-Finish (W2F)	6200	2000	10,400

**Table 3:** Lineage detection by site type.

Site Type	Number of Detections	% Total
Nursery		
L1C.5	2	100
Wean-to-finish		
L1A	5	20.8
L5A	3	12.5
L1C.5	13	54.2
L1D	1	4.2
L1H	2	8.3
Finisher		
L1C.5	4	66.6
L1D	1	16.6
L1H	1	16.6

## Data Availability

The original contributions presented in this study are included in the article/Supplementary Material. Further inquiries can be directed to the corresponding author.

## Supplementary Materials

The following supporting information can be downloaded at: https://www.mdpi.com/article/10.3390/vetsci12101000/s1, Table S1: Bioexclusion practices with *p*-value < 0.02 associated with sites reporting a PRRS outbreak; Table S2: Frequency of bioexclusion practices with *p*-value < 0.05 adopted per site type; File S1: Biosecurity Assessment Survey.
