# Supplementary material for: Assessment of the Relationship Between Bioexclusion Practices Applied in Wean-to-Harvest Sites and PRRS Outbreaks
Source: Vet Sci. 2025 Oct 16;12(10):1000. doi: 10.3390/vetsci12101000 (PMC12568179; doi:10.3390/vetsci12101000)
Supplement: Supplementary file 1 [file vetsci-12-01000-s001.zip › Supplement file/Supplementary material - File S1.pdf]

PROJECT TITLE: INDUSTRY-WIDE ASSESSMENT OF BIOEXCLUSION PRACTICES IN WEAN-TO-HARVEST SITES AND DEVELOPMENT AND VALIDATION OF A RAPID RISK ASSESSMENT BIOEXCLUSION TOOL.

Premises ID ______________________________

Production System ______________________________

Person Completing Survey ______________________________

GPS Coordinates ______________________________

GENERAL INFORMATION

1. Which of this best describes your site? Please select one:
☐ Wean to finish
☐ Nursery
☐ Finishing
2. How many pigs are there on the site?
3. How are pigs on the premise flowed?
☐ All-in all-out by room/barn
☐ All-in all-out by site
☐ Continuous flow
☐ Other (please describe)
Description:
4. Select the stocking density of the site.
☐ Single stocking
☐ Double stocking
☐ Other (please describe)
Description:
5. Ownership/Labor: Select the one that applies to the site.
☐ Producer or production system owns pigs and facilities and provides labor
☐ Producer or production system owns pigs, but facilities and labor are contracted
☐ Producer or production system owns pigs and provides labor, but facilities are contracted
6. Are slow-growing pigs removed from the group prior to the removal of their cohort group?
☐ Yes
☐ No
7. From how many different breeding herds are pigs at the premises sourced?

CHARACTERISTICS OF THE SITE / BIOSECURITY PROTOCOLS

Phase 2: Site-specific long survey to assess Bioexclusion practices.

1. Is the site surrounded by a perimeter fence?
☐ Yes
☐ No
2. Is there a closed gate at all entrances to the premises?
☐ No
☐ Yes, closed and locked at all times
☐ Yes, closed and locked only after hours
☐ Yes, but never locked
3. Are there doors at all entrances to the barns?
☐ No
☐ Yes, closed and locked at all times
☐ Yes, closed and locked only after hours
☐ Yes, but never locked
4. Are other livestock present or adjacent on the site?
☐ Yes
☐ No
5. How many vehicle entrances are there to the premises?
6. Is there an occupied house on the premises?
☐ Yes
☐ No
7. Is there a common vehicle entrance to the barns and residence on the premises?
☐ Yes
☐ No
8. Is there a disinfection/wash area used for vehicles entering the premises?
☐ Yes
☐ No
9. Are there permanent windbreaks (high shrubs, trees) on the premises?
☐ Yes
☐ No
Description:
10. What is the topography of the surrounding area?
☐ Steep hills or mountains
☐ Flat or gentle rolling hills
11. Which of the following best describes your unit perimeter boundaries?
☐ Hedges only
☐ Hedges, trees, shrubs and wire fencing
☐ Stock fencing only

Phase 2: Site-specific long survey to assess Bioexclusion practices.

☐ No boundaries
12. What sanitation and decontamination procedures are used in the facilities on the premises? Mark all that apply.
☐ Always washed between every group
☐ Detergent is used during washing
☐ Disinfectant is used after washing
☐ Always allowed to dry between groups
☐ Minimum of 24 hours downtime between groups
13. How is the site filled?
☐ Room basis
☐ Site basis
14. How many days/weeks does it take to fill the site?
15. What procedures are followed by people (employees, veterinarians, maintenance crew…) when entering barns on the premises? Mark all that apply.
☐ Shower-in required
☐ Shower-out required
☐ Bench entry
☐ Shoe cover station at entry way
☐ Disinfection station at entry way
☐ Hand sanitizer in the entry way
☐ Required to wear boots dedicated to sites
☐ Required to wear clothing dedicated to sites
☐ Hands must be washed or hand sanitizer used prior to entering barns
16. Do the entrance procedures differ between visitors?
17. Is a visitor's log used to record visitors to the premises?
☐ Always
☐ Sometimes
☐ Never
18. Are washing crews used on site?
☐ No
☐ Yes
19. Are vaccination crews used on site?
☐ No
☐ Yes
20. If yes, how many times per lot?

Phase 2: Site-specific long survey to assess Bioexclusion practices.

21. Are load-out crews used on site?
☐ Yes
☐ No
22. What biosecurity procedures are in place for tools, supplies and equipment (eg. cell phones, veterinary equipment, etc.) brought into barns by veterinarians, off-site production personnel, vendors and other visitors? Mark all that apply.
☐ May not be entered
☐ Entered through a dedicated room with a clearly defined clean and dirty side
☐ Decontaminated with disinfectant or other methods before entering the facility
☐ Equipment is restricted to the office area
23. Are vehicles of veterinarians, off-site production personnel, vendors, and other visitors restricted to designated entrances and/or parking areas?
☐ No
☐ Yes
24. What procedures are followed by people (maintenance/repair personnel) NOT entering barns on the premises? Mark all that apply.
☐ Must wear disposable boots or change boots between premises
☐ People and vehicle traffic is purposely routed to avoid crossing paths with personnel and their vehicles on the premises
25. What procedures are in place if people visit or work on other swine premises? Mark all that apply.
☐ Not allowed to visit or work on other swine premises
☐ Minimum of overnight downtime is required
☐ Employee must wash vehicle and disinfect interior before returning to the premises
☐ Members of on-farm employee households are not allowed to be employed by other swine production or swine related operations
26. How biosecurity procedures are managed? Mark all that apply.
☐ SOPs are written in all languages spoken as first language by employees
☐ New employees are formally trained
☐ All employees are periodically retrained
27. How often are needles changed? (Pigs of any age)
☐ Needles are changed only when bent or broken
☐ One needle per room
☐ One needle per pen
☐ Use needle-free device
☐Separate needle for each individual animal
28. Are light/substandard feeder pigs transferred to other premises?
☐ No, they remain with the group.
☐ Yes, they are transferred to another site dedicated to substandard pig

☐ Yes, transferred to another barn in the same site.
29. Are market light/substandard pigs transferred to other premises?
☐ No, they are sold at the end (last cut / barn dump).
☐ Yes, transferred to another site dedicated to substandard pigs.
☐ Yes, transferred to another barn in the same site.

ANIMAL TRANSPORTATION

1. How is/are the trailer(s)/vehicle(s) that hauls pigs to the premises owned and managed?
☐ Dedicated to this premises only
☐ Managed by the producer or production system
☐ Contracted to a 3rd party that hauls exclusively for this production system
☐ Contracted to a 3rd party that hauls swine for other producers or production systems
2. From how many other swine premises may swine be hauled, to or from, by the same trailer(s)/vehicle(s) used to transport pigs? *Mark all that apply.*
Weaned pigs: ☐ same day without washing ☐ same day after washing ☐ after washing and downtime ☐ N/A
Nursery/Feeder pigs: ☐ same day without washing ☐ same day after washing ☐ after washing and downtime ☐ N/A
Market hogs: ☐ same day without washing ☐ same day after washing ☐ after washing and downtime ☐ N/A
Light/Substandard pigs: ☐ same day without washing ☐ same day after washing ☐ after washing and downtime ☐ N/A
3. May PRRSV positive and/or PEDV positive animals ever knowingly hauled on the trailer(s)/vehicle(s) that transport pigs to this premises?
☐ Yes, PRRSV and PEDV positive pigs
☐ Yes, PRRSV positive pigs
☐ Yes, PEDV positive pigs
☐ No
4. Are environmental swabs collected from the trailer(s)/vehicles(s) that transport pigs to this premises and tested for PRRSV and/or PEDV by PCR, after washing/before hauling?
☐ Yes, after every washing
☐ Periodically (Describe interval)
☐ Never
Description:
5. What sanitation and decontamination procedures are used on the trailer(s)/ vehicle(s) that haul pigs to this premises? *Mark all that apply.*
☐ Always washed between every load
☐ Detergent is used during washing
☐ Disinfectant is used during washing

Phase 2: Site-specific long survey to assess Bioexclusion practices.

☐ Always allowed to fully dry naturally before next load
☐ Always dried using a TADD system
☐ Minimum of 24 hours downtime is allowed between loads
☐ Disinfectant is used to decontaminate the floor mats, steering wheel, etc. inside vehicle
6. What biosecurity procedures, training and auditing are done for the truck washes where trailer(s)/vehicle(s) that haul pigs are washed? *Mark all that apply.*
☐ SOPs are written in all languages spoken as first language by employees
☐ New employees are formally trained
☐ All employees are periodically retrained
☐ Compliance with biosecurity procedures are formally audited by a 3rd party
☐ Compliance with biosecurity procedures are formally audited by a party affiliated with the producer or production system
☐ Compliance with biosecurity procedures are formally audited by a party affiliated with the truck wash
7. What biosecurity procedures, training and auditing are done for the truck washes where trucks that haul pigs are washed? *Mark all that apply.*
☐ SOPs are written in all languages spoken as first language by employees
☐ New employees are formally trained
☐ All employees are periodically retrained
☐ Compliance with biosecurity procedures are formally audited by a 3rd party
☐ Compliance with biosecurity procedures are formally audited by a party affiliated with the producer or production system
☐ Compliance with biosecurity procedures are formally audited by a party affiliated with the truck wash
8. How is the unloading area for pigs designed on the premises? *Mark all that apply.*
☐ Bumper-to-bumper transfer done on the premises
☐ Bumper-to-bumper transfer done off the premises
☐ Separate, unattached unloading area on the premises
☐ Attached to buildings housing swine
☐ Chute is washed after every load
☐ Chute is disinfected after every load
9. What biosecurity procedures are followed by the driver of the trailer(s)/vehicle(s) that haul pigs to this premises? *Mark all that apply.*
☐ Wears disposable boots or changes boots between sites
☐ Wears clean or disposable coveralls on-site
☐ Restricted from entering the chute
☐ Restricted from entering the buildings
10. Are cutting boards, whips and other tools used by the driver farm-specific?
☐ Yes

☐ No
11. If Question 12 is a "No", are cutting boards, whips and other tools used by the driver cleaned and disinfected prior to loading the pigs?
☐ Yes
☐ No

DEAD PIGS REMOVAL

1. Are dead animals disposed of on-site?
☐ No
☐ Yes, buried (Skip to Question 5)
☐ Yes, incinerated (Skip to Question 5)
☐ Yes, composted (Skip to Question 5)
☐ Yes, other (describe) (Skip to Question 5)
2. What procedures are followed to prevent virus from a contaminated rendering truck from being transmitted to the herd? *Mark all that apply.*
☐ Dead animal pick-up site is more than 100 yards from swine buildings
☐ Dead animals are stored in an enclosed container
☐ Dead storage area is enclosed by a fence or solid structure
☐ Equipment used to move dead animals from barns to the rendering collection site is dedicated to this premises
☐ Equipment used to move dead animals to the collection site does not cross paths with the rendering truck
☐ People and vehicle traffic are purposely routed to avoid crossing paths with rendering trucks on the premises
☐ Employees are not allowed to re-enter swine barns after moving deads to the collection site the same day
3. How is the rendering truck managed?
☐ Dedicated to this premises only
☐ Managed by the producer or production system
☐ Contracted to a 3rd party that hauls exclusively for this production system
☐ Contracted to a 3rd party that hauls swine for other producers or production systems
4. What procedures are in place to prevent virus from a contaminated rendering truck driver from being transmitted to the herd?
☐ Not allowed to leave truck on the premises
☐ Wears disposable boots or changes boots between premises
5. Where is the on-site dead disposal located relative to swine barns and how is it designed?

6. What are the standard operating procedures for removing dead animals from swine barns at this premises?

FEED/ FEED INGREDIENTS DELIVERY

1. Is there a feed mill on the premises?
☐ Yes
☐ No
2. How is the feed delivery vehicle managed?
☐ Dedicated to this premises only
☐ Managed by the producer or production system
☐ Contracted to a 3rd party that hauls exclusively for this production system
☐ Contracted to a 3rd party that hauls swine for other producers or production systems
3. Does the feed mill have written biosecurity measures in place to reduce the risk of feed becoming contaminated with pathogens?
☐ Yes, for PRRSV
☐ Yes, for PEDV
☐ Yes, for PRRSV and PEDV
☐ No
4. Are feed or feed ingredient deliveries scheduled or routed according to the PRRSV and/or PEDV status of the premises?
☐ Yes, for PRRSV
☐ Yes, for PEDV
☐ Yes, for PRRSV and PEDV
☐ No
5. Are there any downtime requirements for the feed or feed ingredient delivery vehicle before it delivers feed to this premises?
☐ Yes
☐ No
6. What procedures are followed to prevent virus from a contaminated feed delivery vehicle or driver from being transmitted to the herd? *Mark all that apply.*
☐ Driver is not allowed to leave cabin at the premises
☐ Driver wears disposable boots or changes boots between premises
☐ People and vehicle traffic are purposely routed to avoid crossing paths with feed delivery vehicles on the premises
7. How frequently do wild animals have access to feed (i.e. open bags, bin covers left open, spillage that is fed to pigs, etc)? *Mark all that apply.*
Non-migratory birds ☐Weekly ☐ Less than weekly ☐Never
Migratory birds ☐Weekly ☐ Less than weekly ☐Never
Rodents ☐Weekly ☐ Less than weekly ☐Never
Wild animals (racoons, cyotes, etc.) ☐Weekly ☐ Less than weekly ☐Never

Phase 2: Site-specific long survey to assess Bioexclusion practices.

8. What procedures are followed to reduce the risk of contaminated feed or feed ingredients transmitting the virus to the herd? *Mark all that apply.*
☐ Feed is pelleted or heat treated
☐ Chemical or antimicrobial decontamination (formaldehyde, etc.)
☐ No dried distillers' grains (DDGS) in diets
☐ No porcine derived feed ingredients in diets
☐ No animal derived feed ingredients in diets
☐ Spilled feed is sometimes fed to pigs on the premises

PROPANE/FUEL DELIVERY

1. How is the propane/fuel delivery vehicle managed?
☐ Dedicated to this premises only
☐ Managed by the producer or production system
☐ Contracted to a 3rd party that hauls exclusively for this production system
☐ Contracted to a 3rd party that hauls swine for other producers or production systems
2. What procedures are followed to prevent virus from a contaminated propane or fuel delivery vehicle or driver from being transmitted to the herd? *Mark all that apply.*
☐ Tanks located more than 50 yards from swine barns
☐ Driver not allowed inside buildings
☐ Driver wears disposable boots or changes boots between premises
☐ People and vehicle traffic are purposely routed to avoid crossing paths with propane and fuel truck on the premises

GARBAGE COLLECTION

1. How is the garbage truck managed?
☐ Dedicated to this premises only
☐ Managed by the producer or production system
☐ Contracted to a 3rd party that hauls exclusively for this production system
☐ Contracted to a 3rd party that hauls swine for other producers or production systems
2. What procedures are followed to prevent virus from a contaminated garbage truck or driver from being transmitted to the herd? *Mark all that apply.*
☐ Garbage pick-up is located more than 50 yards from swine barns
☐ Driver not allowed inside buildings
☐ Driver wears disposable boots or changes boots between premises
☐ People and vehicle traffic are purposely routed to avoid crossing paths with garbage trucks on the premises

NEW TOOLS/SUPPLIES DELIVERY

1. What procedures are in place to decontaminate new tools or supplies? *Mark all that apply.*
☐ Dedicated room with a clearly defined clean and dirty side through which tools and supplies are entered

Phase 2: Site-specific long survey to assess Bioexclusion practices.

☐ Quarantined for a minimum of 24 hours
☐ Decontaminated with disinfectant or other methods before entering barns
2. What procedures are in place to prevent virus from a contaminated delivery vehicle or driver from being transmitted to the herd? *Mark all that apply.*
☐ All new tools/supplies delivered by on-farm employees
☐ Driver not allowed past a clearly defined clean/dirty line
☐ Driver wears disposable boots or changes boots between premises
☐ Delivery vehicles are restricted to designated entrances or parking areas
**TRANSFERRED TOOLS/SUPPLIES DELIVERY FROM OTHER SITES**
1. What procedures are in place to decontaminate transferred tools or supplies? *Mark all that apply.*
☐ Dedicated room with a clearly defined clean and dirty side through which tools and supplies are entered
☐ UV exposure
☐ Quarantined for a minimum of 24 hours
☐ Decontaminated with disinfectant or other methods before entering barns
☐ N/A no transferred tools
2. What procedures are in place to prevent virus from a contaminated delivery vehicle or driver from being transmitted to the herd? *Mark all that apply.*
☐ All transferred tools/supplies delivered by on-farm employees
☐ Driver not allowed past a clearly defined clean/dirty line
☐ Driver wears disposable boots or changes boots between premises
☐ Delivery vehicles are restricted to designated entrances or parking areas
☐ N/A no transferred tools
**ON-FARM EMPLOYEE MOVEMENT**
1. On an average week day, how many people work on this farm?
2. On an average weekend day, how many people work on this farm?
3. On average, how many times per day do pig care person exit and re-enter the barns?
4. What is the average annual employee turnover at this premises in the last two years?
5. Are part-time employees used?
☐ Weekly
☐ Occasionally
☐ Rarely
☐ Never

Phase 2: Site-specific long survey to assess Bioexclusion practices.

6. What procedures are in place if employees visit or work on other swine premises? *Mark all that apply.*
☐ Not allowed to visit or work on other swine premises
☐ Minimum of overnight downtime is required
☐ Employee must wash vehicle and disinfect interior before returning to the premises
☐ Members of on-farm employee households are not allowed to service other swine production or swine related operations
7. Are on-farm employees allowed to perform other swine related activities (i.e. delivering feed, hauling pigs, etc)?
☐ Yes
☐ No
8. What is the biosecurity, training and auditing protocols for the premises? *Mark all that apply*.
☐ SOPs are written in all languages spoken as first language by employees
☐ New employees are formally trained
☐ All employees are periodically retrained
☐ Compliance with biosecurity procedures are formally audited by a 3rd party
☐ Compliance with biosecurity procedures are formally audited by a party affiliated with the producer or production system
☐ Compliance with biosecurity procedures are formally audited by an independent 3rd party
9. What biosecurity procedures are in place for personal equipment (e.g. Cell phones, watches, etc.) brought into barns by on-farm employees? *Mark all that apply.*
☐ Equipment may not be entered
☐ Equipment is entered through a dedicated room with a clearly defined clean and dirty side
☐ Equipment is decontaminated with disinfectant or other methods before entering the facilities
☐ Equipment is restricted to the office area
10. Does any employee live with an individual that worked on another swine site, visited another swine site, or owns exhibition swine?
☐ Yes
☐ No
11. Do any on-farm employees own other food animals?
☐ Exhibition swine
☐Other livestock
☐ Chicken or other birds
☐ No
12. What are the procedures for entering repairing tool on the site?
☐ Equipment is entered through a dedicated room with a clearly defined clean and dirty side
☐ Equipment is decontaminated with disinfectant or other methods before entering the facilities

☐ No procedures required
13. Do the sanitation procedures for employees exiting and re-entering the barns differ from those when employees first arrive?
☐ Yes
☐ No
14. Are on-farm employee vehicles restricted to designated entrances and/or parking areas?
☐ Yes
☐ No

REPAIR/SERVICE PERSONNEL (INSIDE BARNS)

1. How are repairs that are done inside of swine barns managed?
☐ Dedicated to this premises only
☐ Managed by the producer or production system
☐ Contracted to a 3rd party that hauls exclusively for this production system
☐ Contracted to a 3rd party that hauls swine for other producers or production systems
2. Are entry procedures relaxed when repair personnel exit and re-enter the barns?
☐ Yes
☐ No
3. How much downtime (hours) is required for repair personnel?

REPAIR/SERVICE PERSONNEL (OUTSIDE BARNS)

1. How are repairs that are done outside of swine barns managed?
☐ Dedicated to this premises only
☐ Managed by the producer or production system
☐ Contracted to a 3rd party that hauls exclusively for this production system
☐ Contracted to a 3rd party that hauls swine for other producers or production systems
2. How much downtime (hours) is required for repair personnel?
3. How many times per month is the electrical reader read on this premises? *Mark all that apply.*
☐ What procedures are in place to prevent virus from a contaminated vehicle or driver from being transmitted to the herd when the electrical meter is read?
☐ Electrical meter is located more than 50 yards from swine barns
☐ Driver not allowed inside buildings
☐ Driver wears disposable boots or changes boots between premises
4. How many times per month is the lawn mowed or snow removed?
5. How is lawn mowing managed?

Phase 2: Site-specific long survey to assess Bioexclusion practices.

☐ Mowing equipment is dedicated to this premises and lawn is mowed by on-farm personnel
☐ Managed by the producer or production system
☐ Contracted to a 3rd party that mows exclusively for this production system
☐ Contracted to a 3rd party that mows swine sites for other producers or production systems
How is snow removal managed?
☐ Snow removal equipment is dedicated to this premises and is done by on-farm personnel
☐ Managed by the producer or production system
☐ Contracted to a 3rd party that removes snow exclusively for this production system
☐ Contracted to a 3rd party that removes snow from swine sites for other producers or production systems
**VETERINARIANS/VENDORS/VISITORS/OFF-FARM PERSONNEL**
1. How much downtime (hours) is required for veterinarians, off-site production personnel, vendors, and other visitors?
**PORK/FOOD ENTRY**
1. Are pork/food products prohibited from entering the premises?
☐ Uncooked pork
☐ Processed or cooked pork
☐ Other food/beverages
2. Are pork/food products restricted to the office or lunchroom area of the facilities?
☐ Yes
☐ No
**MANURE REMOVAL**
1. How many times per year is manure or effluent removed from the premises? During which season?
2. How is manure handled and stored at the site?
☐ Deep pit collection and storage under pigs
☐ Shallow pit collection
☐ Outdoor unenclosed lagoon or storage
☐ Outdoor enclosed storage
☐ Flush system using fresh water
☐ Flush system using recycled water
3. What parties are involved with manure or effluent removal from the site?
☐ Manager or on-farm employees
☐ Other personnel employed by producer or production system
☐ 3rd party that removes manure exclusively for the producer or production system
☐ 3rd party that removes manure for other producers or production systems

Phase 2: Site-specific long survey to assess Bioexclusion practices.

4. Are parties involved with manure removal allowed to enter the barns?
5. How is manure removal equipment managed?
☐ Some or all dedicated to this premises
☐ Some or all managed by the producer of production system
☐ Some or all contracted to a 3rd party that removes manure exclusively for this producer or production system
☐ Some or all contracted to a 3rd party that removes manure for other producers or production systems
6. Approximately how many other swine premises is the manure removal equipment used on?
7. Is the manure removal equipment washed and disinfected before entering the premises?
☐ Yes
☐ No
8. How much downtime (hours) is required for manure removal equipment?
9. Are environmental swabs collected from manure removal equipment and tested for PRRSV and PEDV by PCR before entering the premises? *Mark all that apply.*
☐ Always for PPRSV
☐ Always for PEDV
☐ Sometimes for PPRSV
☐ Sometimes for PEDV
☐ Never for PRRSV
☐ Never for PEDV

OTHER ANIMAL ENTRY

1. How frequently are the following types of animals seen on the premises outside of barns? (Weekly, Less than Weekly, Never) *Mark all that apply.*
Feral Swine ☐Weekly ☐ Less than weekly ☐Never
Rodents ☐Weekly ☐ Less than weekly ☐Never
Non-swine domestic animals ☐Weekly ☐ Less than weekly ☐Never
Non-swine wild animals ☐Weekly ☐ Less than weekly ☐Never
Migratory birds ☐Weekly ☐ Less than weekly ☐Never
Non-migratory birds ☐Weekly ☐ Less than weekly ☐Never
2. How frequently are the following types of animals seen on the premises inside of barns? (Weekly, Less than Weekly, Never) *Mark all that apply.*
Feral Swine ☐Weekly ☐ Less than weekly ☐Never
Rodents ☐Weekly ☐ Less than weekly ☐Never
Non-swine domestic animals ☐Weekly ☐ Less than weekly ☐Never

Phase 2: Site-specific long survey to assess Bioexclusion practices.

Non-swine wild animals ☐Weekly ☐ Less than weekly ☐Never
Migratory birds ☐Weekly ☐ Less than weekly ☐Never
Non-migratory birds ☐Weekly ☐ Less than weekly ☐Never
3. Are rodent bait stations used and checked regularly?
☐ Weekly
☐ Monthly
☐ Occasionally
☐ Never
4. In SUMMER months, what is the concentration of insects and/or larvae seen inside of the barns?
☐ Severe
☐ Light to moderate
☐ None
5. In WINTER months, what is the concentration of insects and/or larvae seen inside of the barns?
☐ Severe
☐ Light to moderate
☐ None
6. Is insect control (eg. Insecticide sprays, foggers, baits, etc.) used consistently?
☐ Always
☐ Sometimes
☐ Never

AIR/WATER

1. What is the type of ventilation on the site? Mark all that apply.
☐ Natural
☐ Mechanical
☐ Filtered
☐ Other (Describe)
2. What is the source of drinking water for the pigs? *Mark all that apply*.
☐ Surface waters (lakes, ponds, etc.)
☐ Well
☐ Rural water
☐ Municipal water
3. Is drinking water treated? *Mark all that apply.*
☐ Chlorination
☐ Acidifiers
☐ Iodine
☐ Peroxide
☐ Other

Phase 2: Site-specific long survey to assess Bioexclusion practices.

☐ Drinking water is not treated
