# Supplementary material for: Assessment of the Relationship Between Bioexclusion Practices Applied in Wean-to-Harvest Sites and PRRS Outbreaks
Source: Vet Sci. 2025 Oct 16;12(10):1000. doi: 10.3390/vetsci12101000 (PMC12568179; doi:10.3390/vetsci12101000)
Supplement: Supplementary file 1 [file vetsci-12-01000-s001.zip › Supplement file/Supplementary material - Tables S1 and S2.pdf]

**Table S1.** Description of the bioexclusion practices that had a P-value <0.20 during the univariate analysis.

| Biosecurity practices | Levels | Odds ratio | CI 95% | P-value |
|---|---|---|---|---|
| Dedicated vehicle parking area for staff/visitors | No (REF) | 1 | | |
| | Yes | 0.07 | 0.01–0.35 | 0.001 |
| Dedicated manure pumping equipment per site | No (REF) | 1 | | |
| | Yes | 0.07 | 0.01–0.43 | 0.003 |
| Pigs hauled without confirmed PRRSV status | No (REF) | 1 | | |
| | Yes | 11.18 | 2.17-57.68 | 0.003 |
| Overnight downtime mandatory for multi-farm employee | No (REF) | 1 | | |
| | Yes | 0.15 | 0.04–0.56 | 0.004 |
| Site type | Nursery (REF) | 1 | | |
| | Finisher | 17.47 | 2.44–125.19 | 0.004 |
| | Wean-to-finish | 8.47 | 0.85–84.24 | 0.068 |
| Rodents seen around feed bin | (REF) | | | |
| | Never | 0.08 | 0.02-0.47 | 0.004 |
| Wild animals seen around feed bin | (REF) | | | |
| | Never | 0.08 | 0.02-0.47 | 0.004 |
| Presence of bench entry | No (REF) | | | |
| | Yes | 0.27 | 0.11-0.67 | 0.005 |
| Exclusive 3rd party manure removal | No (REF) | | | |
| | Yes | 0.10 | 0.02-0.53 | 0.006 |
| Rendering | No (REF) | 1 | | |
| | Yes | 6.47 | 1.62–25.84 | 0.008 |
| Allowing employees to cohabitate with others who work swine-related jobs | No (REF) | | | |
| | Yes | 6.15 | 1.51-25.09 | 0.011 |
| Natural vs. mechanical ventilation system | Mechanical (REF) | 1 | | |
| | Natural | 10.88 | 1.35–87.60 | 0.025 |
| Repair outside managed by producer or production system | No (REF) | | | |
| | Yes | 0.26 | 0.08-0.88 | 0.029 |
| Livestock present on the site | No (REF) | | | |
| | Yes | 3.76 | 0.97-14.63 | 0.055 |
| Employees not allowed to work multiple swine premises | No (REF) | | | |
| | Yes | 0.26 | 0.07-1.03 | 0.055 |
| Repair inside barns done by exclusive 3rd party | No (REF) | | | |
| | Yes | 4.50 | 0.91-22.22 | 0.065 |
| Re-entry after repair follows different protocol | No (REF) | | | |
| | Yes | 4.50 | 0.91-22.22 | 0.065 |
| Repair outside barns done by exclusive 3rd party | No (REF) | | | |
| | Yes | 4.50 | 0.91-22.22 | 0.065 |
| Contracted exclusive 3rd party for lawn mowing | No (REF) | | | |
| | Yes | 4.50 | 0.91-22.22 | 0.065 |
| Contracted exclusive 3rd party removes snow | No (REF) | | | |
| | Yes | 4.50 | 0.91-22.22 | 0.065 |
| Number of sites within 3 miles | Reference | | | |
| | Within 3 miles | 1.10 | 0.99-1.23 | 0.078 |

| | | | | |
|---|---|---|---|---|
| Herd size: 5,000–7,500 | Reference Herd size: 5,000–7,500 | 0.11 | 0.01-1.78 | 0.120 |
| New supply to DD room | No (REF)<br>Yes | 0.32 | 0.08-1.41 | 0.135 |
| Average number of employees working on site | Reference | 0.74 | 0.50-1.10 | 0.140 |
| Employee has other production animals | No (REF)<br>Yes | 2.49 | 0.74-8.43 | 0.140 |
| Shoe cover station at entry way | No (REF)<br>Yes | 0.22 | 0.03-1.70 | 0.147 |
| SOPs are written in all languages spoken by employees | No (REF)<br>Yes | 0.22 | 0.03-1.80 | 0.160 |
| Repair tools are decontaminated with disinfectant before entering the site | No (REF)<br>Yes | 0.35 | 0.08-1.56 | 0.169 |
| Employees who work multiple premises must wash vehicle before returning to site | No (REF)<br>Yes | 0.33 | 0.07-1.61 | 0.173 |

*Note: CI denotes confidence interval, representing the range within which the true parameter value is expected to lie with 95% certainty. Reference indicates the base category against which other groups were compared in the regression model.*

**Table S2.** Descriptive statistics (frequency) for the bioexclusion practices with P-value <0.05 by site type during the univariate analysis.

| **Biosecurity practice** | **Levels** | **Nursery** | **Wean-to-finish** | **Finisher** |
|---|---|---|---|---|
| Dedicated vehicle parking area for staff/visitors | Yes | 100.0% | 25.6% | 20.0% |
| | No | 0.0% | 74.4% | 80.0% |
| Dedicated manure pumping equipment per site | Yes | 100.0% | 15.4% | 0.0% |
| | No | 0.0% | 84.6% | 100.0% |
| Pigs hauled without confirmed PRRSV status | Yes | 0.0% | 100.0% | 100.0% |
| | No | 100.0% | 0.0% | 0.0% |
| Overnight downtime mandatory for multi-farm employee | Yes | 100.0% | 64.1% | 100.0% |
| | No | 0.0% | 35.9% | 0.0% |
| Rodents seen around feed bin | Less than weekly | 0.0% | 5.1% | 100.0% |
| | Never | 100.0% | 61.5% | 0.0% |
| | Weekly | 0.0% | 33.3% | 0.0% |
| Wild animals seen around feed bin | Less than weekly | 0.0% | 5.1% | 100.0% |
| | Never | 100.0% | 61.5% | 0.0% |
| | Weekly | 0.0% | 33.3% | 0.0% |
| Presence of bench entry | Yes | 100.0% | 43.6% | 6.7% |
| | No | 0.0% | 56.4% | 93.3% |
| Dead disposal onsite | Yes | 100.0% | 53.8% | 100.0% |
| | No | 0.0% | 46.2% | 0.0% |
| Repair outside managed by producer or production system | Yes | 100.0% | 53.8% | 100.0% |
| | No | 0.0% | 46.2% | 0.0% |
| Natural vs. mechanical ventilation system | Natural | 100.0% | 12.8 | 100.0% |
| | Mechanical | 0.0% | 87.2% | 0.0% |
| Allowing employees to cohabitate with others who work swine-related jobs | Yes | 0.0% | 12.8% | 80.0% |
| | No | 100.0% | 87.2% | 20.0% |
